# Self-report of 24-h urine completeness compared with *para*-aminobenzoic acid (PABA) recovery does not bias estimates of dietary salt intake in the UK

**DOI:** 10.1017/S0007114525106132

**Published:** 2026-01-06

**Authors:** Kerry S. Jones, David Collins, Sarah R. Meadows, Damon A. Parkington, Albert Koulman, Polly Page

**Affiliations:** 1 Nutritional Biomarker Laboratory, MRC Epidemiology Unit, University of Cambridge, Cambridge, UK; 2 Nutrition Measurement Platform, MRC Epidemiology Unit, University of Cambridge, Cambridge, UK

**Keywords:** Sodium, Potassium, 24-h urine, Survey, Paraamino benzoic acid, National Diet, Nutrition Survey

## Abstract

The measurement of sodium excretion in 24-h urine samples is the recommended method to assess dietary salt intake to monitor salt-related public health policies. Ensuring complete collection of 24-h urine samples is important for the accurate assessment of salt intake. We compare the use of the objective biomarker, recovery of *para*-aminobenzoic acid (PABA), to self-reported 24-h urine completeness. Data collected from 868 men and women aged 19–64 years from the England Sodium Survey 2018/2019 (part of the UK National Diet and Nutrition Survey (NDNS)) were used to compare self-reported 24-h urine completeness based on a collection duration of 23–25 h, no missed urine collections/voids and a minimum urine volume of > 0·4 L against completeness based on the urinary recovery of oral doses of PABA. Two-thirds (69 %; 561/812) of participants who adhered to the PABA protocol provided a complete 24-h urine collection. Assessed by self-report, 71% (619/868) of participants provided a complete 24-h urine collection. Sodium excretion was (geometric mean (interquartile range)) 127 (97–170) mmol/24 h with PABA and 126 (97–169) mmol/24 h by self-report; salt intake was 7·40 (5·65–9·94) g/d and 7·38 (4·53–8·83) g/d, respectively. The proportion of participants above the UK-recommended salt intake of 6 g/d was 70 % by both PABA and self-report. This study shows that the use of self-report of 24-h urine collection completeness provides an assessment of sodium excretion and dietary salt intake with the same accuracy as when PABA recovery is used to assess completeness.

High sodium intakes are associated with risk of high blood pressure and greater risk of CVD, whilst reduced salt intake can lower blood pressure and reduce CVD risk^(1,2)^. Consequently, governments have developed public health strategies to lower salt (sodium chloride) consumption with the aim to improve population health^(3)^, including in the UK^(4)^.

The assessment and monitoring of sodium intake are essential to understand population salt intake and the effectiveness of salt reduction programmes^(5)^. Biomarkers of sodium status do not exist due to tight homeostatic control. Dietary assessment of salt intake is known to be inaccurate, partly due to difficulty in recording discretionary salt intake^(6)^. Consequently, the measurement of sodium excretion in urine is the accepted approach to assess sodium/salt intake. Specifically, the use of 24-h urine collections is considered the gold standard, and population salt intakes can be estimated from single collections of 24-h urine samples^(6,7)^. Standard procedures are published by the WHO^(7)^ and European Union^(8)^. Individual assessment of salt intake requires multiple 24-h urine collections^(6)^.

Twenty-four-hour urine collection is timed from the point after a urine void (frequently, but not exclusively the first morning pass) to 24 h later. Ensuring complete collection of 24-h urine samples is important for the accurate assessment of salt intake. Different approaches have been taken to determine completeness. These include a specified window of duration, minimum volume, recording missed collections, calculations based on urinary creatinine and the use of *p*-aminobenzoic acid (PABA). The use of PABA as an objective biomarker to validate complete collection of 24-h urine was first established in 1983^(9)^ and has since been used to determine completeness in clinical studies and population-based studies including sodium surveys of the UK National Diet and Nutrition Survey (NDNS)^(10,11)^. PABA has also been used to verify dietary intake in feeding studies and to assess under- or over-reporting of dietary intake^(12–14)^.

PABA is a small (molar mass = 137 g/mol), naturally occurring molecule and provides an objective marker of 24-h urine completeness. The typical protocol involves participants consuming 3 × 80 mg tablets of PABA evenly spaced starting at the beginning of the 24-h period. Completeness is assessed based on the recovery of PABA. However, there are practical issues with PABA use such as participant compliance and the considerable cost of procurement of PABA tablets and PABA analysis. There are also other known limitations of PABA related to its metabolism and clearance that may challenge the assumptions underpinning its validity. These include differences in metabolism with age, sex, absorptive function and meal timing^(15)^. These differences are manifested in the acceptable range of PABA recovery that constitutes a complete collection. Many studies have considered ≥ 85 % recovery to represent a complete collection, whilst a study from Cox *et al*. concluded that completeness ranged from 75 to 103 % of the expected amount^(16)^. Adjustment of the 24-h urine measurement based on incomplete PABA collection has also been developed and applied^(17,18)^. A few studies have directly compared PABA recovery to other methods to assess completeness, primarily based on cut-offs related to expected creatinine excretion^(19)^. However, the majority of studies^(6)^, including more recent surveys and studies detailed in online Supplementary Table 1, rely on alternative methods to assess completion, including duration of collection, reports of missed collections, urine volume and creatinine excretion. Here, using data from a national survey of salt intake^(10)^, we explore agreement between PABA and self-reported claim to determine the completeness of 24-h urine collection and estimates of salt intake.

## Methods

### Sample size

The available sample was determined from primary data collected as part of the NDNS Sodium Survey (2018/2019) for which the sample size of 565 complete 24-h urine collections was required to detect a 7 % (0·5 g/d) reduction in geometric mean estimated salt intake between the 2014 and 2018/2019 surveys.

### Fieldwork and urine collection

Full details of participant recruitment can be found in the NDNS Sodium Survey 2018/2019 report^(10)^. In summary, participants were sampled from the Health Survey for England (HSE) 2017 cohort^(20)^. Individuals from HSE who consented to be contacted for follow-up research and provided a telephone number were included in the sample. In each household, up to two individuals fulfilling the eligibility criteria were selected to participate.

Biomedical fieldworkers visited participants in their homes and provided the materials to complete the 24-h urine collection. For participants willing to take PABA, they were requested to take the first PABA tablet at the beginning of the 24-h collection period and the following two tablets 4–6 h after the previous tablet. Urine was collected in a 2-litre container and transferred to a 5-litre container until collection by the biomedical fieldworker. Urine was collected by participants for 24 h from the second morning urine pass of the 24-h collection day, until and including the first urine pass the following morning. During the collection period, participants were required to record the time they took the PABA tablets, the start and finish times of their urine collection and any missed urine voids.

Following completion of the 24-h urine collection, a biomedical fieldworker visited the participant in their home. Urine weight was measured with the use of a hanging handheld scale (Brecknell ElectroSamson; Brecknell Scales), recorded in kg. In this report, urine quantity is reported as volume, assuming 1 kg = 1 litre of urine. The biomedical fieldworker transferred urine into 2 × 10 ml conical tubes that were packaged and sent by ambient post to the MRC Epidemiology Unit. On arrival, urine was mixed and transferred into aliquots and frozen at −70°C until analysis.

Further details on all recruitment and fieldwork procedures are published in the relevant NDNS Sodium Survey 2018/2019 report^(10)^.

### Para-aminobenzoic acid analysis

PABA concentration was measured by HPLC at the Nutritional Biomarker Laboratory (NBL) (MRC Epidemiology Unit, University of Cambridge). In the assay, metabolic products of PABA, such as *p*-aminohippuric acid, *p*-acetamidobenzoic acid and *p*-acetamidohippuric acid, are hydrolysed back to PABA under alkaline conditions, and the resultant PABA concentration is determined with UV detection at 290 nm. The PABA HPLC method was based on a previously published method^(21)^ modified to use methanol instead of acetonitrile as the mobile phase^(10)^. Urine samples containing PABA were used for quality control (QC); between-batch imprecision was 4·5 %, 3·7 % and 2·1% at concentrations of 33, 66 and 203 nmol/l, respectively.

### Sodium and potassium analysis

Sodium and potassium were measured using ion-selective electrode technology on a Roche Cobas C111 at the NBL. Accuracy of sodium assessment was determined using commercial calibration materials (HI-7083M and HI-7088M; Hanna Instruments Ltd) traceable to the National Institute of Standards and Technology (NIST) standard reference maternal (SRM). Percent inaccuracy against the target concentrations of prepared solutions was 0·6 % at both 50 and 100 nmol/l. Commercially available QC materials (Urine Chemistry Controls; Bio-Rad Laboratories) were used to determine between-batch imprecision which was less than 2 % for sodium and less than 3 % for potassium. The laboratory participated in the UK National External Quality Assessment Scheme (NEQAS), and the percent bias against the ‘all laboratory trimmed mean’ was −0·2 % for sodium and −4·4 % for potassium.

### 24-h urine completeness

PABA recovery was calculated from the concentration of PABA in the urine sample and urine volume, and excretion was calculated as a percentage of the 240 mg PABA dose given to participants. A urine collection was considered complete if the PABA recovery was between 70 and 103 %^(10,16)^.

For completeness by self-reported claim, the following criteria were applied: (1) urine volume > 0·4 litres, (2) no reports of missed collections and (3) collection duration of 23–25 h. We also investigated the same criteria but with an increased acceptable duration of between 22 and 26 h (online Supplementary Table 2).

### Salt intake

Sodium concentration measured in mmol/l and total urine volume were used to calculate 24-h sodium excretion. From this, daily salt intake was calculated based on 17·1 mmol of sodium being equal to 1 g of salt^(10)^.

### Statistics

Statistics were performed in Stata 17 (StataCorp) and GraphPad Prism (GraphPad Software). Variables with normally distributed data are presented as mean and standard deviation and skewed data as geometric mean with lower and upper quartiles. Although these data are those collected as part of the NDNS Sodium Survey 2018/2019, the average salt intakes reported in this present analysis are not the same as the reported survey results which were 7·5 g/d, 6·8 g/d and 8·3 g/d (geometric means) for the total population, women and men, respectively^(10)^. This is because (i) the data reported here are not weighted for survey response and (ii) the criteria for assessing 24-h urine completeness are not the same since NDNS also included participants who had taken less than three PABA tablets but reported no missing collections and had a collection duration of 23–25 h.

Cohen’s kappa was used to assess agreement between the methods and used a dataset restricted to those who had taken three PABA tablets, resulting in a dataset of 805 participants.

Multiple linear regression was used to investigate predictors of PABA recovery in urine. Parameters included were sex, age, duration of collection, missed collections (yes/no) and urine volume.

## Results

**Figure 1:**
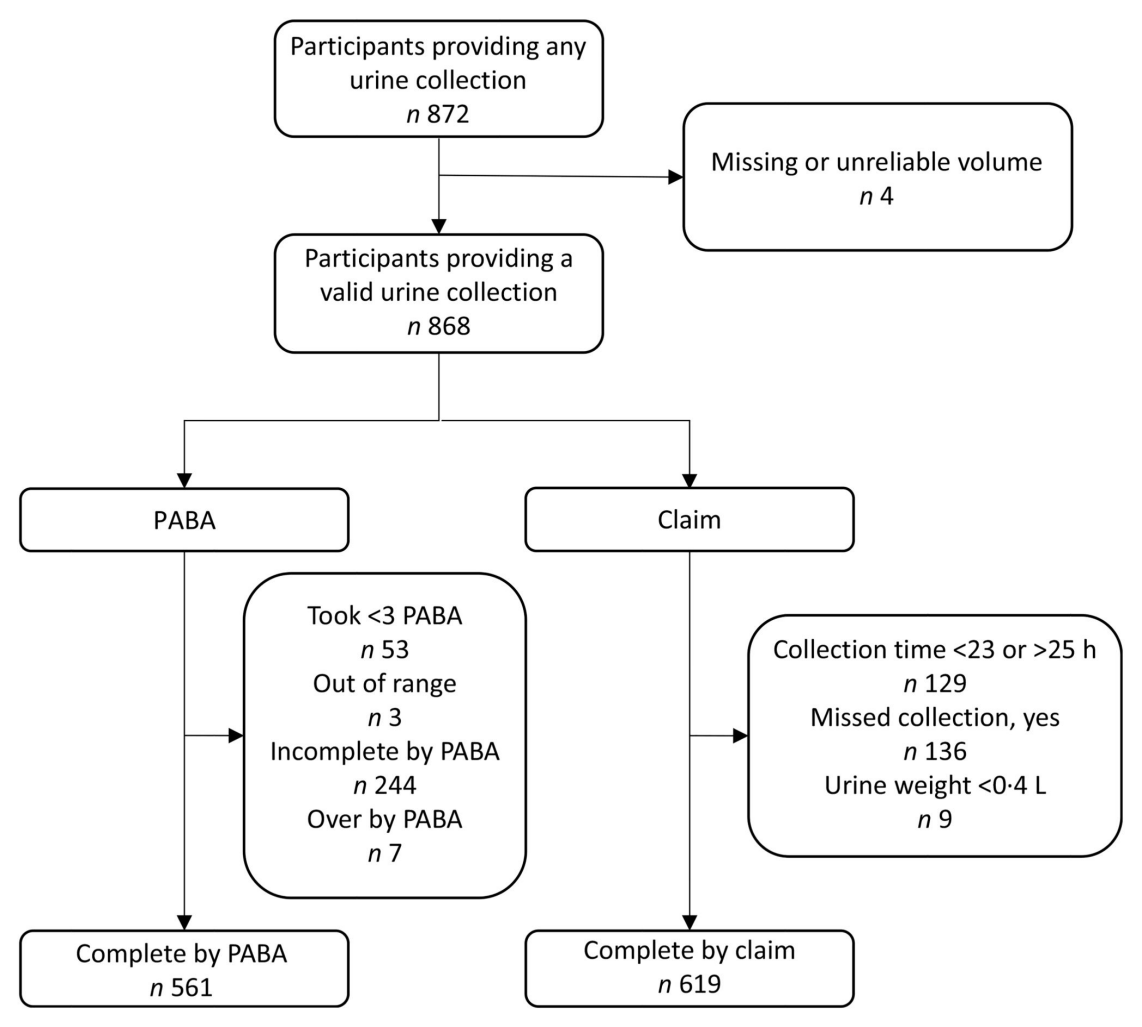
Flow chart of participants with complete 24-h urine samples determined either by PABA recovery or participant claim and included in the analysis of salt intake. *Some participants were excluded for more than one reason, and hence the categories of exclusions sum to more than total exclusions. PABA, *para*-aminobenzoic acid.

Urinary sodium concentrations were available from 872 men and women aged 19 to 64 years, of whom four were excluded due to missing or unreliable urine volume data. Figure 1 shows the inclusion and exclusion criteria as a flow chart to compare the different methods to assess the completeness of the 24-h urine collection. Three PABA tablets were consumed by 812 participants, of which 561 (69 %) were deemed to have provided a complete 24-h urine collection. For the assessment of completion by self-reported claim, a total of 868 participants were considered, of whom 619 were assessed to have provided a complete 24-h urine collection. Nine participants had a urine volume of less than 0·4 litres, and the remaining excluded participants were broadly split between duration of collection that was out of range and missed urine collections.

### Participant characteristics, sodium excretion and salt intake

The age of participants was similar in each approach. Proportionally more females provided an incomplete collection than males. Urine volume was higher for complete collections, whether completeness was assessed by PABA or claim. Sodium and potassium excretion were similar for complete collections, whether completeness was assessed by PABA or claim. Sodium excretion was 127 mmol/24 h with PABA and 126 mmol/24 h by claim, and the spread of values (indicated by the interquartile range) was unaffected by the choice of approach to assess completeness. Salt intake was 7·40 and 7·38 g per d, respectively, for collections complete by PABA and complete by claim, with similar distribution of values. The proportion of participants above the WHO-recommended salt intake of 5 g/d was 83·2% and 83·5% by PABA and claim, respectively (Table 1). Whether PABA or claim, either method provided discrimination between complete and incomplete 24-h urine collections for salt intake. Further relaxation of criteria for completeness by claim, specifically by increasing the allowable duration to 22–26 h, resulted in a geometric mean salt intake of 7·32 g, slightly lower than when completeness by claim used the smaller window of 23–25 h (7·38 g). Full results for the 22–26 h criteria are reported in online Supplementary Table 2.

**Figure 2:**
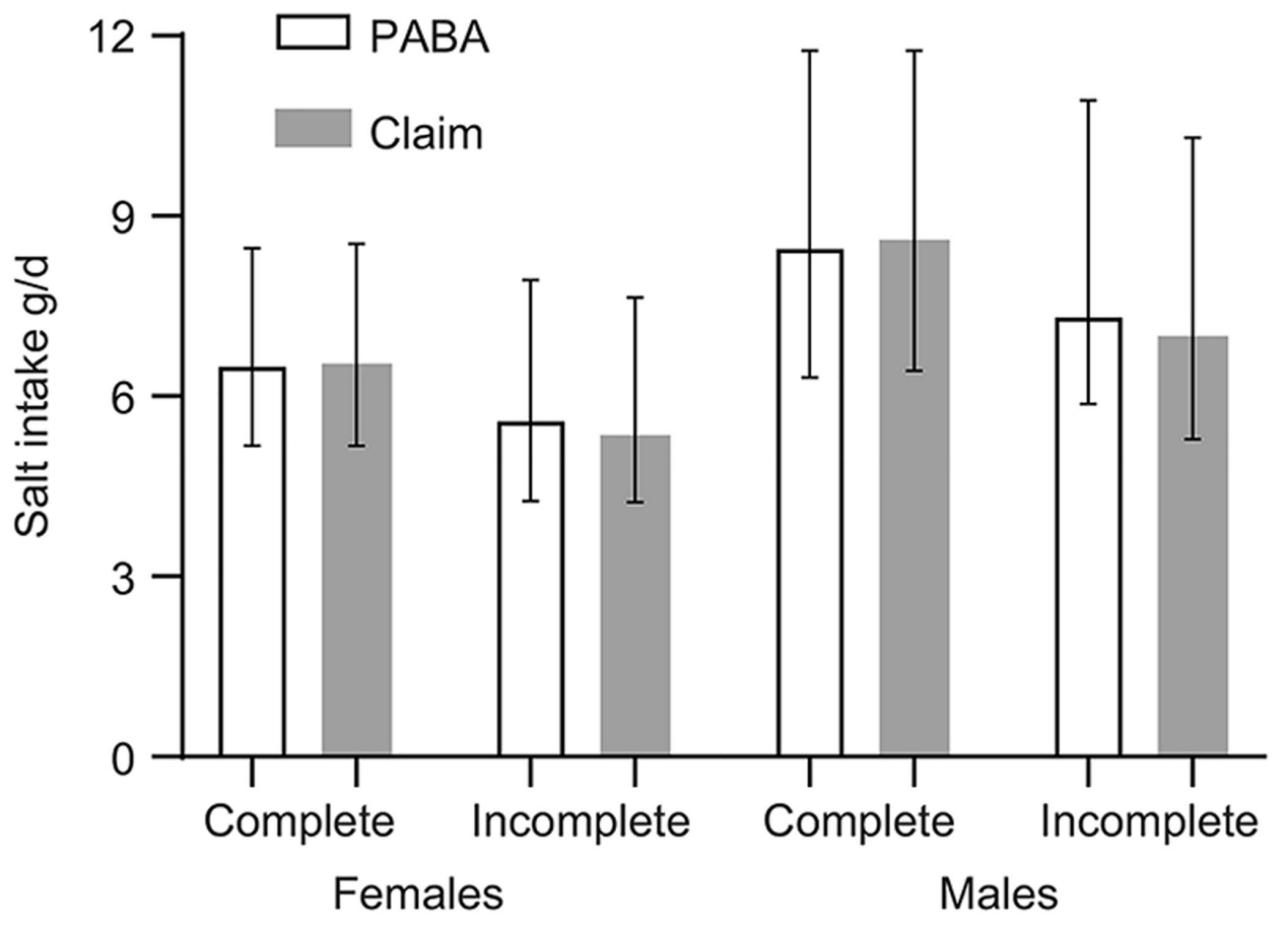
Salt intake in males and females with 24-h completeness assessed by PABA recovery and self-reported claim (see text for details). Bars indicate geometric mean and error bars the interquartile range. PABA, *para*-aminobenzoic acid.

Sodium excretion was higher in males than females (online Supplementary Tables 3 and 4). Whilst salt intake was similar between methods for females (6·51 *v*. 6·54 g/d for complete by PABA and complete by claim, respectively), there was a larger difference between methods for males (8·47 *v*. 8·61 g/d) (Figure 2).

When assessed by claim, a larger percentage of females than males (31% *v*. 26%) provided an incomplete sample. Of those samples considered incomplete by claim, for females 66% had a missed collection whereas for males the figure was 36%.

### Agreement between methods

Cohen’s kappa indicated slight agreement on completion of the 24-h samples between methods (*k* 0·17; *P* < 0·00001); however, overall agreement was 66 %. Of samples judged complete by PABA, 75 % were also judged complete by claim. Agreement for incomplete collections was lower; of samples incomplete by PABA, 43 % were judged incomplete by claim (Table 2). When the sexes were considered separately, overall agreement was lower for females (62 %) than males (70 %).

### Determinants of para-aminobenzoic acid recovery

Overall, multiple linear regression significantly predicted PABA recovery in females (*P* = 0·0001), males (*P* = 0·01) and for the sexes combined (*P* < 0·0001). In the sexes combined model, sex (*β*-coefficient (95% CI), 3·6% (1·3, 5·9); *P* = 0·002) and ‘missed collections’ (*β*-coefficient, −8·8% (−11·9, −5·7); *P* < 0·0001) were both significant predictors of PABA recovery, although all the tested variables together only explained 6% (adjusted R-squared) of the variation in PABA recovery. When further analysed by sex, for females ‘missed collections’ (*β*-coefficient, −8·5% (−12·3, −4·7); *P* < 0·0001) and urine volume (*β*-coefficient, 1·6% (0·09, 3·2); *P* = 0·04) were significant predictors (adjusted R-squared, 4%) and for males only ‘missed collections’ (*β*-coefficient, −9·6% (−15·1, −4·2); *P* = 0·001) (adjusted R-squared, 3 %). There was no indication of any relationship between age and PABA recovery.

## Discussion

Accurate assessment of population daily salt intake by sodium excretion in urine relies on a complete 24-h urine collection. Using data collected as part of the UK NDNS, we showed that estimated population daily salt intake was similar whether the completeness of 24-h urine collection was assessed objectively through the use of PABA recovery or through self-reporting of urine collection duration and missed collections.

A systematic review from 2016 investigated the accuracy of other methods relative to PABA to assess the completeness of 24-h urine^(19)^. Criteria for completeness included creatinine excretion, self-report of missing urine and volume, but the authors found no one method was significantly more accurate than another, compared with PABA. In the review, whilst sodium excretion was found to vary by completion status regardless of the completion criteria, the direction of difference was inconsistent and the magnitude was small; overall, the authors concluded that estimates of sodium excretion may not be biased by inclusion of participants with incomplete 24-h urine collection^(19)^. Knuiman *et al*.^(15)^ reported that 75–85 % of about 180 participants in eleven European countries who claimed they had an incomplete collection had PABA recovery below the study cut-off, suggesting that self-report of incomplete collections was reliable. Conversely, only 12 % of women and 22 % of men who self-reported complete collection had PABA recovery below the cut-off^(15)^. By way of comparison, in the present study, 43 % of participants claiming incomplete collection were also considered incomplete by PABA (49 % in females and 34 % in males). In 681 adult participants from a study in Canada, agreement between self-reported completeness and PABA was about 60 %, similar to our study, and sodium excretion was also found to be similar (3577 mg/d *v*. 3643 mg/d for self-report and PABA recovery, respectively). Subar *et al*.^(22)^ compared PABA recovery to self-report of missing collections during 24-h urine collections in about 350 participants measured on two occasions in the USA. Restricting analysis to only participants who took PABA, 86 % of those who reported no missed voids were judged complete by PABA. In our study, 75 % of the participants who took PABA were judged to have a complete sample. Estimates of protein and potassium intake and variation were similar, whether completeness was inferred from PABA recovery or self-report^(22)^. Of note, both Knuiman^(15)^ and Fu^(23)^ observed that agreement between completeness assessed by PABA recovery and creatinine excretion was relatively poor; Fu also found better agreement between PABA recovery and self-reported completeness^(23)^. Differences in agreement between PABA recovery and self-reported completion will in part vary due to differences in methods to assess self-reported completion (e.g. the specific questions asked) as well as the level of participant engagement.

Other studies have suggested that PABA recovery performs best when monitoring compliance over a number of days because within- and between-person variation in PABA excretion is high when used over a single 24-h period^(13,22)^. As a consequence of high variation, there is a relatively large range of PABA recovery that is considered to indicate a complete 24-h collection, anywhere between 70 and 110 %^(16,19)^. This increases the chance of including specimens that are not complete since an individual may have collected only 70 % of their urine.

It is essential to understand the limitations of the PABA approach. First, in the majority of studies included in the review of John *et al*.^(19)^, PABA concentration was measured by the colorimetric assay rather than the more specific HPLC technique^(21)^. Inter-individual differences in the pathways and rates of PABA metabolism as well as an effect of age^(24,25)^ and absorption due to meal timing^(15)^ may also affect PABA recovery. In contrast, we and other studies^(23)^ did not observe an effect of age, possibly because the age range of participants of our study was restricted to a maximum of 64 years. A further limitation of using PABA recovery alone is that it is not possible to determine over-collection, that is, collection above 24 h. In addition, although naturally occurring amounts of PABA in the diet are low, an online search suggests that PABA can be found in supplements in the UK market, typically in amounts ranging from 20 to 30 mg in multivitamins to over 400 mg in some niche products, and these could impact the accuracy of the PABA method to assess completeness.

As may be expected, in regression analysis, PABA recovery was predicted by missed collections. However, this is also encouraging since it demonstrates a degree of engagement and compliance from participants and shows that their report of collection completeness is largely accurate. The data presented here also showed that sex and urine volume were significant predictors of PABA recovery. Whilst their contribution was relatively minor, where completeness of collection is apparently borderline, these variables may further contribute to the uncertainty around defining completeness based on PABA recovery. Sex differences in PABA recovery may be attributed to sex differences in pharmacokinetics and metabolism^(26)^ and/or the practicalities of urine collection. Duration of collection was not a significant predictor of PABA; studies relying on self-reporting of collection completeness have used different durations ranging from 22 h or more^(27)^, 23 to 25 h^(28)^, to 22 to 26 h^(29)^ or 20 to 28 h^(30)^. The latter study also normalised urine concentrations to the collection period, adjusting urinary sodium concentration to 24 h.

Despite limited agreement between PABA recovery and self-reported completeness observed in this study, estimates of salt intake were similar for each approach, a finding in keeping with earlier studies^(15,19,22,23)^. This suggests that population estimates of sodium and salt intake may be relatively robust when different criteria are used to define the individuals included in a population sample. Whilst agreement between identification of incomplete *v*. complete collections was not perfect, the study supports, as described above, other published evidence demonstrating that the method of assessing completeness may not bias estimates of salt intake on the population level.

Use of PABA in 24-h urinary sodium studies incurs additional costs, logistical challenges and participant burden. In a 2014 review that included national and subnational population studies of sodium intake using urinary biomarkers to assess salt intake^(19)^, of those that used 24-h urine, only two of eighteen surveys (England and Scotland) used PABA recovery to assess completeness of the urine collection. Since then, other than from the UK, there have been no reports of the use of PABA in large population surveys (including the USA^(27)^ and China^(29)^). Other large multicentre population studies (INTERSALT and INTERMAP) did not use PABA and relied partly on self-report, but the start and end of the collection period were performed at an examination centre under supervision^(31,32)^. In 2022, a systematic review of data describing salt intake in all European countries reported that twenty-two out of fifty-three studies used 24-h urine collections, of which three used PABA^(33)^: the UK, Ireland and a small study from the Czech Republic. The use of PABA tends to be restricted to smaller and more detailed studies often for the validation of dietary questionnaires or alternative methods to estimate salt intake^(17,34–36)^. Furthermore, the use of PABA for population urinary sodium surveys is not given as a recommendation in the conclusions from a recent report of the International Consortium for Quality Research on Dietary Sodium/Salt (TRUE consortium)^(37)^ and is stated as not recommended by the WHO^(7)^. Our findings from this comparison study of data collected in the NDNS Sodium Survey (2018/2019) support these recommendations. These findings are also relevant to other biomarkers that benefit from measurement in 24-h urine, for example, in relation to markers of dietary intake, including non-nutritive sugars^(38,39)^.

Despite the relatively large sample size and PABA and sodium urine concentration data from all participants regardless of completion status, our study has a number of limitations. It is unclear if the use of PABA in the study affected participant behaviour and compliance with reporting of collection completeness. Data categorising missed collections was obtained from a single question; thus, it was not possible to distinguish between a complete missed collection (i.e. of substantive urine volume likely to impact the calculation of individual salt intake) and, for example, a few drops of missed urine likely to have no or minimal impact. Other studies of 24-h sodium excretion have posed more detailed questions, for example, in a feasibility study of 24-h urine collection in the US National Health and Examination Survey, seven questions were asked about the amounts, frequency and circumstances of missed collections^(40)^. The use of exclusions based on creatinine excretion per kg body weight or equations to adjust sodium excretion based on estimated to measured creatinine excretion^(41)^ was not possible because weight, height and ethnicity of the participants were not collected. The majority of population surveys of sodium intake collect anthropometric data that allow calculation of creatinine excretion. Whilst providing an adjunct estimate of completeness or for sensitivity analysis, evidence from the studies reviewed above suggests that completeness based on creatinine cut-offs are no more accurate than other methods. The study did not include older adults (65+ years); therefore, limiting the possibility of comparing PABA recovery between younger and older age groups. Whilst these data support the use of self-report to assess 24-h urine completeness in a survey population or large study setting, this approach may not be adequate for smaller, metabolic-type studies such as to ensure compliance with an intervention. Furthermore, multiple 24-h urine collections are required to accurately estimate individual salt intakes^(42,43)^; thus, no inferences can be made about the comparison of the two approaches for individuals.

## Conclusions

Using data from a national survey, estimates of population salt intake in complete 24-h urine collections were similar whether urine collection completeness was assessed by PABA recovery or by participant self-report. The advantages of using self-report include lower participant burden, lower costs and fewer excluded participants. Future studies of 24-h urine collection to estimate population salt intake should aim to standardise completeness criteria and questions related to self-reporting of completeness to allow pooling of data, although country context considerations may make this difficult. Overall, this study shows that the use of self-report of 24-h urine collection completeness provides an assessment of sodium excretion and dietary salt intake with the same accuracy as when PABA recovery is used to assess completeness.

## Supplementary Material

For supplementary material/s referred to in this article, please visit https://doi.org/10.1017/S0007114525106132.

1

## Figures and Tables

**Table 1:** Participant and urinary excretion data and salt intake by completion method of PABA recovery or claim (self-report of collection duration and missed collections)*

	Complete by PABA recovery								Complete by claim (23-25 h, no missed collections, urine volume > 0-4 litres)						
	Complete *n* 561				Incomplete *n* 244				Complete *n* 619				Incomplete *n* 249		
	Mean		SD		Mean		SD		Mean		SD		Mean		SD
Age (years)	48·4		10·6		47·8		11·4		48·6		10·8		47·2		11·0
Sex (%female)		51·5				69·7				56·1				62·2	
Collection hours (h)	23·9		0·8		24·0		0·9		24·0		0·4		23·8		1·6
Missed collections (years)															
*n*		56				67				0				136	
%		10%				27%				0%				55%	
Urine volume (litres)	2·34		1·03		246		1·10		2·31		1·00		2·13		1·12
PABA recovery (%)	82·4		74		544		15·0		76·3		16·7^2^		69·7		18·4^†^
Sodium (mmol/l)															
Geometric means		59·6				55·8				60·5				56·0	
Lower, upper quartiles		41·1, 85·0				39·6, 78·2				41·9, 85·0				37·8, 83·4	
Potassium (mmol/l)															
Geometric means		35·4				344				35·7				35·0	
Lower, upper quartiles		26·9, 45·6				264, 444				26·8, 46·4				26·7, 45·5	
Creatinine (mmol/l)	6·05		1·67		5·63		1·63		6·03		1·65		5·92		1·72
Sodium (24 h mmol)															
Geometric means		127				104				126				101	
Lower, upper quartiles		97, 170				77, 148				97, 169				77, 151	
Potassium (24 h mmol)															
Geometric means		75·1				644				74·9				63·3	
Lower, upper quartiles		60·5, 94·2				51·5, 874				60·4, 95·0				50·8, 84·9	
Creatinine (24 h mmol)	12·8		1·34		10·5		1·57		12·6		1·37		10·7		1·57
Salt intake (g/d)															
Geometric means		740				647				7·38				5·92	
Lower, upper quartiles		5·65, 9·94				4·51, 846				5·67, 9·91				4·53, 8·83	
> 5 g/d^‡^ (%)		83·2 %				71·3 %				83·5 %				67·9 %	
> 6 g/d^‖^ (%)		69·5 %				57·0 %				69·6 %				54·2 %	

PABA, *para*-aminobenzoic acid.

* Age, collection hours, urine and PABA recovery are mean (SD). Sodium, potassium and salt are geometric means and lower and upper quartiles.

† Only participants where three PABA tablets were taken.

‡ WHO-recommended salt intake is < 5 g/d.

‖ UK-recommended salt intake is < 6 g/d.

**Table 2:** Agreement between 24-h urine collection completeness by PABA recovery or claim

		Completeness by claim								
		Complete			Incomplete			Total		
		*n*	%		*n*	%		*n*		%
Completeness by PABA	Complete	431	74·6		130	57·3		561		69·7
	Incomplete	147	254		97	42·7		244		30·3
Total		578	100		227	100		805		100

PABA, *para*-aminobenzoic acid.

## Abbreviations

NDNS: National Diet and Nutrition Survey
PABA: para-aminobenzoic acid
