# Supplementary material for: Self-report of 24-h urine completeness compared with para-aminobenzoic acid (PABA) recovery does not bias estimates of dietary salt intake in the UK
Source: Br J Nutr. Author manuscript; Available in PMC 2026 Feb 27. (PMC7618804; doi:10.1017/S0007114525106132)
Supplement: 1 [file EMS211883-supplement-1.docx]

**Supplementary material**

**Self-report of 24-hour urine completeness compared to PABA recovery does not bias estimates of dietary salt intake in the UK**

Kerry S. Jones, Dave Collins, Sarah R. Meadows, Damon A. Parkington, Albert Koulman, Polly Page

**Supplementary Table 1. Recent studies of salt intake with 24 h urine collection and method to assess urine completeness**

| Study / Reference / Publication year | Completeness criteria | Total collected, n (complete, n) | Breakdown of excluded participants |
| --- | --- | --- | --- |
| NDNS (UK) Sodium Survey (2018/19)  Ashford et al., 2020 ^(1)^ | - by PABA: - duration 20 – 28 h - PABA recovery 70 – 103% - if no PABA then by claim: - duration 23 – 25 h - no missed collections | 897 (596) | n=1 lost in post  n=24 duration OOR  n=7 discrepant data  n=269 incomplete |
| NHANES (USA), 2014  Cogswell et al., 2018 ^(2)^ | - recorded start and stop times - 22 h or more duration - total urine volume of ≥400 mL - reported no more than a few drops of urine lost during collection - no reported menstruation during collection | 1,014 [Phase 1]  (827) | n=120: did not finish  n=5: duration <22 h  n=52: missed urine  n=5: volume <400 ml  n=5: other |
| Sub-sample of the Barbados Health of the Nation (HotN) cross-sectional study  Harris et al., 2018  ^(3)^ | - volume not <500 mL or >5000 mL - duration 20 to 28 h - not >1 missing collection - sensitivity analysis: UrC (1) excluded if 24 h UrC was <4 mmol in women and <6 mmol in men. (2) based on expected UrC excretion and 0.7 cut-offs | 368 (364) | n=4: volume OOR |
| Community-Based Management of Non-Communicable Diseases in Nepal (COBIN) cohort  Neupane et al, 2020 ^(4)^ | - urine volume ≥500 mL - estimated daily UrC excretion was >6 mmol for men or >4 mmol for women - self-reported spillage of <30 mL | 499 (451) | n=48 |
| Italy, national cross-sectional studies  Donfrancesco et al., 2021 ^(5)^ | - urine volume ≥500 mL - excluded if UrC excretion outside the range of population mean ± 2 SD | 2008-12  (1,858)  2018-2019  (1,977) | 2008-12  n=122  2018-2019  n=104 |
| WHO-SAGE wave 3 (2018–2019)  South Africa & Ghana  Menyanu et al., 2021 ^(6)^ | - Urine volume >300 mL - UrC excretion >4 mmol/day (women) or >6 mmol/day (men) | South Africa: 1,189 (707)  Ghana: 1,121 (495) | NR |
| EH-UH 2 survey (Epidemiology of arterial hypertension and salt intake in Croatia), a nationally representative survey of non-institutionalised  persons in Croatia  Glavic et al., 2024 ^(7)^ | - recorded start and end times - >22 h and <26 h duration - urine volume ≥500 mL - not menstruating - no more than a few drops of urine were missed during collection - UrC -body weight within sex-specific distribution (5.9–26.0 mmol/24 h for men and 4.0–16.4 mmol/24 h for women) | 1,268 (1,067) | n=52 <500 ml  n=97 UCr OOR |
| Nationally representative sample of the adult population of Slovenia (2022)  Kugler et al, 2024 ^(8)^ | - urine volume ≥500 mL - ≤ 1 missed void - >23 h and <25 h duration - delivery from collection finish to lab <24 h - the 24 h UrC concentration was outside ± 2 SD for sex-specific distribution | 568 (518) | n=14 outside duration  n=4 >1 missed void  n=4 <500 mL  n=28 UCr OOR |
| China National Nutrition Survey, 2015  Liu et al., 2024 ^(9)^ | - start and end times that matched the time logic - duration 22 to 26 h - urine volume not <400 mL - urine loss less than a few drops during collection - no pregnancy or menstruation   24-h UrC excretion suitable for Chinese people (7.1–17.7 mmol/24 h for males and 5.3–15.9 mmol/24 h for females) | 17,566  (10,114) | n=397 <22 h  n=400 >26 h  n=336 missed urine  n=272 <400 mL  n=165 ‘polluted’  n=54 menstruation  n=5,828 UCr OOR |

Abbreviations: NR, not report; OOR, out of range; PABA, para-aminobenzoic acid; UCr, urinary creatinine;

References for Supplementary table 1.

1. Ashford R, Jones KS, Collins D, Earl K, Moore S, Koulman A *et al.* (2020) *National Diet and Nutrition Survey. Assessment of salt intake from urinary sodium in adults (aged 19 to 64 years) in England, 2018 to 2019*.

2. Cogswell ME, Loria CM, Terry AL, Zhao L, Wang C-Y, Chen T-C *et al.* (2018) Estimated 24-Hour Urinary Sodium and Potassium Excretion in US Adults. *JAMA* **319**, 1209–1220.

3. Harris RM, Rose AMC, Hambleton IR, Howitt C, Forouhi NG, Hennis AJM *et al.* (2018) Sodium and potassium excretion in an adult Caribbean population of African descent with a high burden of cardiovascular disease. *BMC Public Health* **18**, 998.

4. Neupane D, Rijal A, Henry ME, Kallestrup P, Koirala B, Mclachlan CS *et al.* (2020) Mean dietary salt intake in Nepal: A population survey with 24-hour urine collections. *J Clin Hypertens (Greenwich)* **22**, 273–279.

5. Donfrancesco C, Lo Noce C, Russo O, Minutoli D, Di Lonardo A, Profumo E *et al.* (2021) Trend of salt intake measured by 24-h urine collection in the Italian adult population between the 2008 and 2018 CUORE project surveys. *Nutr Metab Cardiovasc Dis* **31**, 802–813.

6. Menyanu E, Corso B, Minicuci N, Rocco I, Zandberg L, Baumgartner J *et al.* (2021) Salt-reduction strategies may compromise salt iodization programs: Learnings from South Africa and Ghana. *Nutrition* **84**, 111065.

7. Marinović Glavić M, Bilajac L, Bolješić M, Bubaš M, Capak K, Domislović M *et al.* (2024) Assessment of Salt, Potassium, and Iodine Intake in the Croatian Adult Population Using 24 h Urinary Collection: The EH-UH 2 Study. *Nutrients* **16**. doi:10.3390/nu16162599.

8. Kugler S, Blaznik U, Rehberger M, Zaletel M, Korošec A, Somrak M *et al.* (2024) Twenty-four hour urinary sodium and potassium excretion in adult population of Slovenia: results of the Manjsoli.si/2022 study. *Public Health Nutr* **27**, e163.

9. Liu Z, Man Q, Li Y, Yang X, Ding G, Zhang J *et al.* (2024) Estimation of 24-hour urinary sodium and potassium excretion among Chinese adults: a cross-sectional study from the China National Nutrition Survey. *Am J Clin Nutr* **119**, 164–173.

**Supplementary Table 2. Participant and urinary excretion data and salt intake by completion method of PABA recovery or claim (self-report of collection duration [22 to 26 h] and missed collections) in the NDNS Sodium Survey 2018/19^1^**

|  | Complete by PABA recovery | | Complete by claim (22 – 26 h, no missed collections, urine volume >0.4 L) | |
| --- | --- | --- | --- | --- |
|  | Complete  n=561 | Incomplete  n=244 | Complete  n=696 | Incomplete n=172 |
| Age, y | 48.4 (10.6) | 47.8 (11.4) | 48.6 (10.7) | 46.5 (11.3) |
| Sex, %female | 51.5 | 69.7 | 55.2 | 68.6 |
| Collection hours | 23.9 (0.8) | 24.0 (0.9) | 23.9 (0.6) | 23.9 (1.7) |
| Missed collections, yes | 56 (10%) | 67 (27%) | 0 (0%) | 136 (79%) |
| Urine weight, kg | 2.34 (1.03) | 2.16 (1.10) | 2.29 (1.00) | 2.13 (1.19) |
| PABA % | 82 (7) | 54 (15) | 76 (17)^2^ | 68 (19)^2^ |
| Sodium mmol/L | 59.6 (41.1, 85.0) | 55.8 (39.6, 78.2) | 60.3 (41.4, 85.2) | 53.8 (37.9, 79.0) |
| Potassium mmol/L | 35.4 (26.9, 45.6) | 34.4 (26.1, 44.6) | 35.7 (26.9, 46.3) | 34.7 (26.7, 45.8) |
| Creatinine, mmol/L | 6.05 (1.67) | 5.63 (1.63) | 6.05 (1.65) | 5.75 (1.75) |
| Sodium, 24 hr mmol | 127 (97, 170) | 104 (77, 148) | 125 (96, 169) | 94 (71, 147) |
| Potassium, 24 mmol | 75.1 (60.5, 94.2) | 64.0 (51.5, 87.1) | 74.2 (60.0, 94.0) | 60.8 (47.8, 84.5) |
| Creatinine, 24 mmol | 12.8 (1.34) | 10.5 (1.57) | 12.6 (1.37) | 10.1 (1.63) |
| Salt intake, g/d | 7.40 (5.65, 9.94) | 6.07 (4.51, 8.66) | 7.32 (5.62, 9.90) | 5.51 (4.21, 8.62) |

1 Age, collection hours, urine and PABA recovery are mean (SD). Sodium, potassium and salt are geometric means and lower and upper quartiles.

2 Only participant where 3 PABA tablets were taken

**Supplementary Table 3. Participant and urinary excretion data and salt intake by completion method of PABA recovery or claim (self-report of collection duration and missed collections) for females in the NDNS Sodium Survey 2018/19^1^**

|  | Complete by PABA recovery | | Complete by claim (23 – 25 h, no missed collections, urine volume >0.4 L) | |
| --- | --- | --- | --- | --- |
|  | Complete  n=289 | Incomplete  n=170 | Complete  n=347 | Incomplete n=155 |
| Age, y | 48.3 (10.5) | 46.9 (11.4) | 48.3 (10.8) | 46.2 (11.1) |
| Collection hours, h | 23.9 (0.8) | 24.0 (0.9) | 24.0 (0.4) | 23.9 (1.4) |
| Missed collections, y | 38 (13%) | 51 (30%) | 0 (0%) | 102 (66%) |
| Urine weight, kg | 2.27 (0.98) | 2.09 (1.00) | 2.21 (0.92) | 2.07 (1.07) |
| PABA recovery, % | 82 (7) | 55 (15) | 75 (18)^2^ | 68 (19)^2^ |
| Sodium, mmol/L | 54.0 (37.5, 76.3) | 52.5 (38.6, 73.1) | 55.1 (38.7, 74.0) | 52.0 (36.7, 77.0) |
| Potassium, mmol/L | 32.9 (25.7, 43.2) | 33.1 (25.4, 42.1) | 33.2 (25.9, 42.8) | 33.4 (25.4, 43.8) |
| Creatinine, mmol/L | 5.08 (1.62) | 5.14 (1.54) | 5.19 (1.58) | 5.18 (1.64) |
| Sodium, 24 hr mmol | 111 (88, 145) | 96 (73, 136) | 112 (88, 146) | 92 (72, 131) |
| Potassium, 24 mmol | 67.8 (53.8, 84.8) | 60.3 (47.2, 81.1) | 67.5 (54.2, 84.9) | 58.8 (45.5, 81.6) |
| Creatinine, 24 mmol | 10.5 (1.23) | 9.36 (1.52) | 10.5 (1.28) | 9.1 (1.53) |
| Salt intake, g/d | 6.51 (5.17, 8.46) | 5.60 (4.26, 7.93) | 6.54 (5.17, 8.54) | 5.35 (4.23, 7.64) |

1 Age, collection hours, urine and PABA recovery are mean (SD). Sodium, potassium and salt are geometric means and lower and upper quartiles.

2 Only participant where 3 PABA tablets were taken

**Supplementary Table 4. Participant and urinary excretion data and salt intake by completion method of PABA recovery or claim (self-report of collection duration and missed collections) for males in the NDNS Sodium Survey 2018/19^1^**

|  | Complete by PABA recovery | | Complete by claim (23 – 25 h, no missed collections, urine volume >0.4 L) | |
| --- | --- | --- | --- | --- |
|  | Complete  n=272 | Incomplete  n=74 | Complete  n=272 | Incomplete n=94 |
| Age, y | 48.4 (10.7) | 49.8 (11.1) | 48.9 (10.8) | 48.9 (11.0) |
| Collection hours, h | 23.8 (0.8) | 24.1 (0.9) | 24 (0.4) | 23.5 (1.9) |
| Missed collections, y | 18 (7%) | 16 (22%) | 0 (0%) | 34 (36%) |
| Urine weight, kg | 2.42 (1.08) | 2.33 (1.28) | 2.42 (1.09) | 2.24 (1.19) |
| PABA recovery, % | 83 (7) | 53 (16) | 78 (15)^2^ | 73 (18)^2^ |
| Sodium mmol/L | 66.2 (43.3, 102.8) | 64.2 (48.1, 97.4) | 67.3 (46.1, 100.4) | 63.4 (40.0, 112.3) |
| Potassium mmol/L | 38.2 (28.5, 52.4) | 37.6 (28.8, 52.8) | 39.1 (28.8, 53.0) | 37.8 (28.6, 53.5) |
| Creatinine, mmol/L | 7.28 (1.63) | 6.97 (1.72) | 7.30 (1.64) | 7.38 (1.72) |
| Sodium, 24 hr mmol | 145 (108, 201) | 125 (100, 187) | 147 (110, 201) | 120 (90, 177) |
| Potassium, 24 mmol | 83.7 (69.0, 104.2) | 73.4 (59.2, 98.5) | 85.5 (70.9, 106.7) | 71.3 (60.5, 95.6) |
| Creatinine, 24 mmol | 15.9 (1.23) | 13.6 (1.50) | 16.0 (1.25) | 13.9 (1.43) |
| Salt intake, g/d | 8.47 (6.31, 11.75) | 7.33 (5.87, 10.92) | 8.61 (6.42, 11.75) | 7.00 (5.28, 10.3) |

1 Age, collection hours, urine and PABA recovery are mean (SD). Sodium, potassium and salt are geometric means and lower and upper quartiles.

2 Only participant where 3 PABA tablets were taken
